# Trends in Body Mass Index among Icelandic Adolescents and Young Adults from 1992 to 2007

**DOI:** 10.3390/ijerph7052191

**Published:** 2010-05-04

**Authors:** Sigríđur Þ. Eiđsdóttir, Álfgeir L. Kristjánsson, Inga D. Sigfúsdóttir, Carol E. Garber, John P. Allegrante

**Affiliations:** 1 Department of Health and Behavior Studies, Teachers College, Columbia University, 525 West 120th Street, New York, NY 10027, USA; E-Mail: ste2105@columbia.edu; 2 Icelandic Centre for Social Research and Analysis, School of Health and Education, Reykjavik University, Ofanleiti 2, 103 Reykjavík, Iceland; E-Mails: alfgeir@ru.is (A.L.K.); ingadora@ru.is (I.D.S.); 3 Department of Biobehavioral Sciences, Teachers College, Columbia University, 525 West 120th Street, New York, NY 10027, USA; E-Mail: ceg@2140columbia.edu; 4 Department of Sociomedical Sciences, Mailman School of Public Health, Columbia University, 722 West 168th Street, New York, NY 10032, USA

**Keywords:** adolescents, body mass index, Iceland, obesity, overweight, prevalence

## Abstract

Trends in body mass index (BMI) among 51,889 14- to 20-year-old Icelandic adolescents and young adults were examined using data from cross-sectional population surveys conducted from 1992 to 2007. Prevalence of overweight increased for both genders in all age groups, except for 14- and 20-year-old girls. Obesity prevalence increased among boys in all age groups, except for 16-year-olds, and among 15- and 20-year-old girls. The largest increase in obesity rates among both genders was found in the oldest age group. Moreover, not only has the prevalence of obesity increased, but also the extent of obesity has grown more severe among 15- and 17-year-olds boys and among girls in the oldest age group.

## 1. Introduction

Globally, the prevalence of overweight and obesity has increased among children, adolescents, and adults [1]. Being overweight or obese is a growing public health threat because of its significant contribution to the burden of chronic diseases, including type 2 diabetes, cardiovascular disease, hypertension, and some types of cancers [1,2]. Aside from human suffering, the economic consequences that accompany the morbidity and mortality associated with obesity are estimated to contribute up to 6% of the total healthcare costs in many of the European and other developed countries, with indirect costs in lost productivity adding equally as much [1,3].

A critical developmental period for the onset of obesity is during adolescence and young adulthood [4,5]. Although the onset of obesity is less associated with morbidity in adolescence and young adulthood, it is a strong determinant of obesity and affiliated morbidity later in life, with 50% to 80% of obese adolescents growing into obese adults [6]. Adolescent onset of overweight is also associated with adult obesity-affiliated morbidity [7,8], independent of adult weight status [9]. Thus, from a public health perspective, monitoring the prevalence and trends in overweight and obesity among adolescents and young adults is of increasing importance. Furthermore, adolescents and young adults who are overweight or obese are often not only burdened by decreased capacities for physical activity [10], but they may also experience profound psychosocial complications that can affect their social interactions and school performance [11–13].

Upward trends in overweight and obesity among adolescents and young adults have been documented around the globe [14,15], and in most of the Nordic counties [16–19]; recent reports have noted stabilization in the rates of adolescent and childhood overweight and obesity from countries such as Australia [20], the United States [21], and France [22]. However, few data are available on national trends in BMI among Icelandic adolescents [19], and there are limited published studies examining changes over time in both under- and normal-weight adolescents.

In order to tailor prevention programs more effectively, a rigorous examination of the trends in adolescent onset of obesity and general trends in weight change among this age group is needed. But describing only the overweight and obese proportion of the population, or only the central part of the upper-weight distribution, provides an insufficient picture of the current situation [19,23]. In doing so, the entire distribution of body mass index (BMI) may be shifted upward, or a subgroup of the population may be seen as heavier now than before, with little change in the remainder of the population [24]. Thus, it is important to examine the shape of the whole distribution. Moreover, although prevalence rates portray the distribution of BMI, they do not provide important information about the severity of the health problem, or whether the normal, overweight, and obese are becoming heavier.

The aim of this study was to examine the trends in BMI distribution among 14- to 20-year-old Icelandic adolescents and young adults over time and within four separate weight categories during the period from 1992 to 2007. In addition, we sought to compare temporal trends in BMI in Iceland with those observed in other countries.

## 2. Methods and Procedures

This study utilized population data from the national surveys of Icelandic adolescents, *Youth in Iceland*. These comprise a series of cross-sectional surveys that are conducted annually to understand trends in attitudes, values and behavior of youth along with social circumstances and background. The findings are used to enhance public understanding of youth lifestyles and to inform programs that can promote health and social well-being of Icelandic young people. The data analyzed in this study were collected in upper secondary schools at four time periods for 14- and 15-year-olds, in 1992, 1997, 2000, and 2006; and for three time periods for 16- through 20-year-olds in high-schools in 1992, 2004, and 2007. In Iceland, schooling is mandatory for youth age 6 through 15 and about 95% of upper secondary school graduates continue their education in high-schools, which are funded by the municipalities but supervised and operated by the Ministry of Education. High school typically requires about four years to complete, usually by age 19 or 20; however, the proportion of graduates that continue in high school falls to about 85% by the second year and slightly below 70% by the 4th and final year [25]. The distribution of the student population in Iceland is consistent with the distribution of the general population, with about 63% in the capital city, Reykjavik, and surrounding areas.

### 2.1. Procedure

The data collection in the *Youth in Iceland* surveys was guided by a strict methodological protocol [26]. It is carried out by the Icelandic Centre for Social Research and Analysis (ICSRA) at Reykjavik University’s School of Health and Education in cooperation with the Icelandic Ministry of Education, Science, and Culture, the municipalities around the country, and the vast majority of Iceland’s schools. All aspects of data collection were approved by an Icelandic central human subjects review committee, and a passive consent was required. The ICSRA administered the distribution of anonymous questionnaires to all upper secondary and high-schools in Iceland. Teachers at individual school sites assisted the students with their participation in the study and supervised the survey questionnaire returns. All students who were present in school on the survey day completed their questionnaire inside their classrooms. Upon completion, students placed and sealed the questionnaire in blank envelopes. In 1992 and 1997, the surveys were conducted by the predecessor to the ICSRA, the Institute for Educational Research, using the same protocol. Tables 1 and 2 provide information on the number of participants and response rates within each respective survey.

In total, 24,707 participants completed the four surveys in upper secondary schools.

In total, 27,182 participants completed the three surveys in high schools.

### 2.2. Filtering of Insufficient Information

In upper secondary schools, those respondents who provided insufficient information about gender, age, height or weight, or reported their weight as less than 30 kg and/or above 120 kg, were excluded from the analysis. This resulted in a total number of 22,519 participants (91% of the participants on whom data were available across all four survey years).

Among high-school students, those who reported being below or above the 16- to 20-years-of-age group were excluded from the analysis as were unrealistic weight and height values. Those reporting to be above 170 kg in weight and either under 130 cm or above 220 cm in height were therefore excluded from the analysis as well. The total number of participants in high schools numbered 21,318 (78% of whom provide data across the three survey years).

### 2.3. Measure and Classification of BMI Groups

Self-reported height and weight were used to calculate BMI (kg/m^2^). For young adults, 18 years old and above, BMI was categorized according to the World Health Organization [1] classification as follows: underweight ≤ 18.5 kg/m^2^, normal weight = 18.5–24.9 kg/m^2^, overweight = 25–29.9 kg/m^2^, and obese ≥ 30 kg/m^2^. For adolescents under 18 years of age, the International Obesity Task Force (IOTF) age- and gender-specific cut-off points for underweight, overweight and obese (based on centile curves defined to pass through the BMI of 18.5, 25 and 30 kg/m^2^ at age 18) were used to provide internationally comparable estimates of the prevalence among adolescents [27,28]. As recommended by the IOTF [28], overweight was defined as the proportion of adolescents between overweight and obese, so the overweight group does not include the obese group. Because the data were collected in March, the BMI cut-off point values for half years, 14.5, 15.5, were used for upper secondary school students. Data collection among high-school students was conducted in October, thus whole-year cut-off values were used.

### 2.4. Analysis

**Figure 1.**
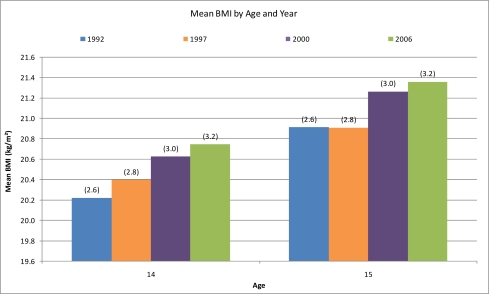
Mean BMI (kg/m^2^) of 14- and 15-year-old Icelandic adolescents in 1992, 1997, 2000, and 2006 (SD in parentheses).

**Figure 2.**
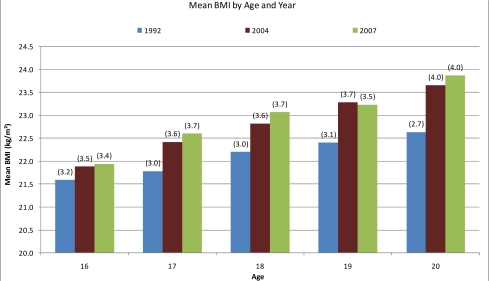
Mean BMI (kg/m^2^) of 16- to 20-year-old Icelandic adolescents in 1992, 2004 and 2007 (SD in parentheses).

Trends in average BMI between age groups and year of study are reported in Figures 1 and 2. We conducted a Chi-square test for trends in prevalence rates of underweight, normal weight, overweight and obesity within each respective age and gender group. The results are reported in Tables 3 and 4. We also conducted a linear trend analysis using Analysis of Variance (ANOVA) for gender- and age-specific mean changes within BMI categories. These are reported in Tables 5, 6, and 7. After considering the distributional properties of the data with the normality assumptions in using ANOVA in mind, we transformed the distribution of the data with natural logarithmic calculations. As a result Skewness and Kurtosis changed from 1.26 and 2.87 to 0.64 and 1.05 respectively.

## 3. Results

Mean BMI increased consecutively over the study period for both genders across all age groups. As shown in Figures 1 and 2, the increase was most profound among 17- to 20-year-olds. Mean BMI for both 14- and 15-year-olds increased by 0.5 kg/m^2^ from 1992–2006 as shown in Figure 1. The same trend was observed among16- to 20-year-olds. From 1992 to 2007, average BMI increased between 0.4–1.2 kg/m^2^ respectively, for the 16-, 17-, 18-, 19- and 20-year-olds. The biggest increase was found in the oldest age group.

### 3.1. Under- and Normal Weight

As the first columns in Tables 3 and 4 reveal, there were no statistically significant changes in the proportion of underweight in any age group for either gender, while a significant reduction in rates of normal weight was detected across all age groups for both genders, except from the 14- and 18-year-old girls. However, the changes in rates were slightly u-shaped for the 19-year-old boys, and 16-, 19-, and 20-year-old girls. The biggest decline in normal weight was found among 20-year-old boys, a reduction from 84.2% to 61.1%, and the most notable shift in girls was also among the 20-year-olds, a decline from 78.9% in 1992 to a 68.0% in 2004, and up to 71.2% in 2007.

### 3.2. Overweight and Obesity

As shown in the third columns in Tables 3 and 4, there was a statistically significant increase in overweight during the study period among boys in all age groups and among girls in all age groups, except for the 14- and 20-year-olds. The greatest increase was found among boys in the oldest age group, an increase from 14.6% to 27.6%. Further, obesity rates increased significantly among boys in all age groups, except for 16-year-olds, and among 15 and 20-year-old girls. The biggest change in obesity rates among both genders was found in the oldest group; from 0.8% to 8.5% among the boys and from 2.6% in 1992 up to 9.2% in 2004 and down to 6.3% in 2007 among the girls.

### 3.3. Within Each Weight Category

As the first columns in Tables 5, 6, and 7 show, there were no statistically significant trends in the underweight category apart from the 15-year-old girls, where the average BMI decreased slightly, or by 0.2 kg/m^2^ over the study period. In the normal-weight category, small but statistically significant increases of 0.1, 0.2, and 0.3 kg/m^2^, were observed among 14-, 15-, and 19-year-old boys, respectively, while a 0.2 kg/m^2^ u-shaped decrease, from 22.5 down to 22.1 and up to 22.3 kg/m^2^ in 2007, was observed among the 20-year-old boys. Similarly, among girls, small but statistically significant increases of 0.4, 0.2, and 0.2 kg/m^2^ were found among the 17-, 18-, and 19-year-olds, respectively. In the overweight category a significant increase in average BMI were observed among 16- and 20-year-old boys and 17-year-old girls, in whom BMI increased by 0.4, 0.6, and 0.5 kg/m^2^, respectively. In the obese category, there was an increase in average BMI for boys in all age groups but only statistically significantly so among the 15- and 17-year-olds, where the average BMI increased by 0.9 and 2 kg/m^2^, respectively. In contrast, among girls in the obese category there was a slight decrease in average BMI across all age groups, but only significantly so among the 19-year-olds, by 1.8 kg/m^2^, with the exception of 20-year-old girls, who experienced a mean increase of 3 kg/m^2^.

## 4. Discussion

This is the first study to present national trend and prevalence data for underweight, normal weight, overweight, and obese Icelandic adolescents and young adults. Our results show a clear shift away from normal weight status. The findings reveal an increased prevalence of overweight between 1992 and 2006/2007 for both genders in all age groups, except for 14- and 20-year-old girls. Obesity prevalence also increased among boys in all age groups, except for 16-year-olds, and among 15- and 20-year-old girls. Moreover, not only did the prevalence of obesity increase, but the extent of obesity grew more severe among 15- and 17-year-olds boys and among girls in the oldest age group. These results are worrisome because health-care costs rise more sharply with higher BMI in the obese than the normal-weight population [29], and recent projections suggest a reduction in life expectancy will occur as a result of this [30].

Our results are also particularly striking in light of several recent reports that have shown a plateau in the prevalence of overweight and obesity among adolescents [20–22]. However, a parallel trend has been reported among the Icelandic adult population from 1990 to 2007, where the rate of obesity in males rose from 7.2% to 18.9% and from 9.5% to 21.3% in females, with 66.6% of males and 53.3% of females being overweight and obese in 2007 [31]. Rankings among 17 European countries showed overweight and obesity levels above 20% in seven countries among adolescents aged 13 to 17 years [32]. England reached the highest prevalence point at 25%, followed by, Italy, Cyprus, Ireland, Greece, Bulgaria, and Spain. Denmark and Hungary reported overweight and obesity levels of 15% to 20%, while Poland and Finland reported rates of 10 to 15% among this age group [32]. In comparison, our findings display an 18.4% prevalence of overweight and obesity among 14- to 17-year-olds in 2006/2007; however, as the survey years differ, the comparison requires some caution when interpreting [32].

Our results also display an unfavorable pattern of overweight and obesity with increased age. Among adolescents from 14- to 20-years-old, the prevalence of being overweight and obese increased from 21.8% to 36.1% among boys and from 12.4% to 25.8% among girls, with more than one-third of 20-year-old boys and over one-fourth of 20-year-old girls being overweight or obese in 2007. Our findings point to the threat to health of the population, whose impact is likely to grow even more serious because parental obesity—a known risk factor for childhood obesity [33]—is increasing concurrently and obesity in children and adolescents is linked to poor health outcomes in adulthood [7–9]. Additionally, the prevalence of rising obesity in older-adolescent and young-adult age groups will likely result in a higher number of pregnant women being overweight or obese. Obese mothers are much more likely to have children with high birth weights [34], which, in turn, can set the stage for obesity later in life; as shown in the cohorts born in Iceland in 1988 and 1994, children who weighed above the 85th percentile at birth were more likely than others to be overweight at the ages of 6, 9, and 15 years [35].

According to an Icelandic dietary consumption survey, young people—especially young men—dine less at home with their families than in previous years, and they also have the highest rates of fast-food, prepared-meal, and sugar consumption in the population [36]. Physical inactivity is an important determinant of overweight throughout the western world [37,38], even after controlling for dietary patterns [37], and it may play an important role in the trends observed in the BMI distribution in this study. In a recent study of the same population over the same time period, a 6% increase in the rate of vigorous physical activity was observed, with a 15% increase in active sports club participation from 1992 to 2006 among 14- to 15-year olds [39]. However, despite these levels of reported activity, over half of the adolescents did not achieve the recommended levels of participation in physical activities. Furthermore, there was also an overall increase in the proportion of inactive adolescents over time, with girls consistently reporting higher levels of physical inactivity compared with boys, even though the net increase in physical inactivity was higher for boys over this time period [39]. This trend is consistent with the clearly increasing prevalence of overweight and obese boys found in this study. Although the number of health clubs, recreational facilities, and organized leisure-time physical activities have all increased over time, there is a paradoxical decrease in the levels of daily physical activity experienced in daily lives.

Taken together, our findings suggest that Iceland may be facing an increasing burden due to chronic diseases associated with overweight and obesity. As the severity of overweight and obesity increases, so do the risks of adult health problems [40–42]. Childhood obesity is associated with an increase in metabolic abnormalities, including insulin resistance, type 2 diabetes mellitus, dyslipidemia, hypertension and polycystic ovary syndrome that begin in childhood and continue into adulthood [43]. In adults, the health consequences of obesity can be particularly devastating. For example, a one-unit increase in BMI is associated with an increased risk of heart failure at the rate of 5% in adult men and 7% in adult women [44], and is associated with a 6% increase in the relative risks for ischemic and hemorrhagic stroke [45]. Adverse health consequences occur not only among those who are in overweight and obese categories; disease risk starts to increase even for those at the upper end of the normal range (BMI of between 22 and 24.9) [41,44]. For example, it is recommended that adults maintain a BMI between 18.5 and 21.9 to minimize their risk of disease [44] because more than three-quarters of the cases of type 2 diabetes are attributable to BMI just exceeding 21 kg/m^2^ [45].

### 4.1. Study Strengths and Limitations

This study has several strengths. First, high-quality data were obtained from a large, representative population cohort with a high response rate. Second, the data contain several age groups, which enabled us to avoid the overgeneralization of adolescent trends over multiple age groups. Third, the survey items and data collection methods were consistent across the study years; and the timing of the studies, sampling, and data collection methods were identical throughout the study period. Fourth, given the mandatory nature of the Icelandic school system for the 14- and 15-year-olds and the high proportion of the 16- to 20-year-olds who continue their education into high-schools, in spite of the fact that schooling is not mandatory, the data collected for these age groups still encompass 70–80% of the total population in this age group. Therefore, it can be assumed that the few missing data (either from absences or improperly completed surveys) were likely randomly distributed and do not reflect a selected sub-category of the study population. Finally, this study not only identifies overweight and obesity prevalence rates but also the relative changes in pattern in underweight and normal weight and within each weight category. To date, most studies have only looked at the prevalence rates of overweight and obesity but have not focused on changes over time in the distribution of BMI, nor in the mean BMI within weight categories.

There are two limitations worth noting. First, although BMI is generally accepted as a valid indicator of body composition for purposes of population-level assessment [1,24], using BMI as an index of adiposity poses some challenges. The limitations of using BMI include inaccurate weight classifications due to high muscle-to-fat ratios, variability in the timing and tempo of adolescent growth spurt, and sexual maturation, all of which are potentially confounding factors [46]. Second, the use of self-reported height and weight data can be problematic, as it is known that some adolescents may tend to over-report their height, and underreport their weight; this is likely to be the case among the overweight and obese [47]. Thus, BMI values based on self-reported height and weight can under-estimate the true prevalence of overweight and obesity. However, other studies have found high correlations between measured and self-reported estimates of adolescent height and weight [17], indicating that self-reported overweight and obesity prevalence rates may be valid. For example, a Norwegian study showed that the sensitivity and specificity of self-reported BMI for identifying overweight among school children were 83% and 100%, respectively [17]. Finally, unless the pattern of misreporting has changed between the survey years, the possible bias from self-reports should be the same over the measurement points and therefore not affect the overall findings.

### 4.2. Implications for Practice and Future Research

The causes of the observed increase in overweight and obesity prevalence are likely influenced by the interaction of several elements, including genetic, behavioral, psychological, and environmental factors. Changes in environmental attributes seem to be the most critical category responsible for the increase in overweight and obesity, since it would be difficult to argue for such a rapid change in genetic composition. Increases in energy consumption, decreasing energy expenditure, or a combination of both can result in energy imbalances and thus lead to increases in weight [48]. Recent social and environmental trends are only likely to affect weight levels negatively. These include a shift toward diets characterized by increased intakes of high-energy density foods, in tandem with trends toward decreased physical activity due to the increasingly sedentary nature of daily life [1,48].

Our results, combined with evidence about the health consequences of obesity in adolescence and adulthood, indicate that immediate actions are needed to reverse the observed trend. For adolescents, particularly among boys, the overall BMI distribution has shifted upward, suggesting that few Icelandic adolescents and young adults are immune to the environmental influences that contribute to significant weight gain. Consequently, intervention approaches need to be broad but population-specific, focusing on ecological factors and changes in the social and environmental determinants of youth lifestyles. Measures should be directed at settings in the society where conditions for healthy dietary habits and physical activity can be created in order to make adoption of healthy lifestyle both feasible and relatively easy. Further, although preventive efforts are more effective than treating established obesity [15], our findings suggest that community-wide preventive efforts also need to be matched by effective treatments for weight loss and weight maintenance.

## 5. Conclusions

The percentage of 14- to 20-year-old Icelandic adolescents and young adults who are overweight increased from 1992 to 2007, except among 14- and 20-year-old girls. The prevalence of obesity increased among boys in all ages, except for 16-year-olds, and among 15- and 20-year-old girls. For the adolescents, particularly among the boys, the BMI distribution has shifted upward, indicating that few Icelandic adolescents and young adults are immune to the ecological factors that appear to account for the observed increase in weight gain that is now being seen globally. Our findings highlight the seriousness of the increasing threat posed by the obesity problem in Iceland.

## Figures and Tables

**Table 1.** Number of 14- to 15-year-olds in compulsory secondary schools who participated in the Youth in Iceland surveys, 1992, 1997*, 2000, and 2006.

Year	N	% of Population	% Males
1992	7,018	85	50
1997	3,913	45	52
2000	6,346	82	49
2006	7,430	82	50

* The sample in 1997 was split in two groups (sample group A and sample group B) for methodological purposes. Questionnaires were randomly distributed to students in both groups. In the current study, we use the data from sample group A with a total of 3,913 responses.

**Table 2.** Number of non-compulsory high-school students who participated in the Youth in Iceland surveys, 1992*, 2004, and 2007.

Year	N	% of eligible population	% Males
1992	4,922	--	49
2004	11,031	81	48
2007	11,229	72	48

* In 1992 the survey was based on a random sample. The eligible population is an estimate of the proportion of all day-school students that normally attend school and is based on information from the high schools.

**Table 3.** Prevalence rates of underweight, normal weight, overweight, and obesity among 14- and 15-year-olds in 1992, 1997, 2000, and 2006.

		Boys		Girls	
BMI category	Year	14	15	14	15
Under	1992	3.9	3.1	10.3	8.9
	1997	3.5	3.3	9.5	8.9
	2000	3.5	2.5	9.1	9.7
	2006	4.2	3.8	9.7	9.2

Chi-square		χ^2^(3,5460) = 1,31, p = 0.726	χ^2^(3,5656) = 4.52, p = 0.210	χ^2^(3,5574) = 1.18, p = 0.758	χ^2^(3,5829) = 0.75, p = 0.862

Normal	1992	83.7	82.6	80.2	81.5
	1997	79.7	82.6	78.8	80.2
	2000	77.4	77.4	78.1	77.6
	2006	74.1	75.9	77.9	76.5

Chi-square		χ^2^(3,5460) = 45.28, p = 0.000	χ^2^(3,5656) = 32.38, p = 0.000	χ^2^(3,5574) = 3.15, p = 0.369	χ^2^(3,5829) = 15.05, p = 0.002

Over	1992	11.6	13.2	8.5	8.5
	1997	15.4	12.0	10.3	9.4
	2000	16.1	17.4	10.8	10.9
	2006	18.1	16.2	10.4	11.8

Chi-square		χ^2^(3,5460) = 26.97, p = 0.000	χ^2^(3,5656) = 19.27, p = 0.000	χ^2^(3,5574) = 5.55, p = 0.136	χ^2^(3,5829) = 11.68, p = 0.009

Obese	1992	0.8	1.0	1.0	1.1
	1997	1.4	2.1	1.5	1.5
	2000	3.0	2.8	2.0	1.7
	2006	3.7	4.1	2.0	2.5

Chi-square		χ^2^(3,5460) = 35.64, p = 0.000	χ^2^(3,5656) = 32.91, p = 0.000	χ^2^(3,5574) = 6.04, p = 0.109	χ^2^(3,5829) = 9.52, p = 0.023

**Table 4.** Prevalence rates of underweight, normal weight, overweight, and obesity among 16- to 20-year-olds in 1992, 2004, and 2007.

		Boys					Girls				
BMI category	Year	16	17	18	19	20	16	17	18	19	20
Under	1992	3.2	3.8	2.5	1.4	0.4	6.4	7.9	10.5	8.6	6.0
	2004	3.9	4.1	3.6	3.5	2.1	8.2	8.5	7.7	5.4	6.0
	2007	3.4	4.1	4.0	2.8	2.4	7.7	8.4	7.4	6.8	2.9

Chi-square		χ^2^(2,3079) = 0.70, p = 0.704	χ^2^(2,2536) = 0.09, p = 0.955	χ^2^(2,2154) = 2.00, p = 0.369	χ^2^(2,1797) = 4.09, p = 0.129	χ^2^(2,833) = 3.55, p = 0.169	χ^2^(2,3155) = 1.47, p = 0.481	χ^2^(2,2700) = 0.22, p = 0.896	χ^2^(2,2371) = 4.85, p = 0.088	χ^2^(2,2127) = 4.91, p = 0.086	χ^2^(2,721) = 2.86, p = 0.239

Normal	1992	80.4	81.4	84.7	81.4	84.2	82.8	80.2	77.4	79.8	78.9
	2004	72.4	71.1	73.6	67.9	69.2	76.6	74.6	73.8	70.8	68.0
	2007	71.1	69.9	70.1	68.2	61.6	77.6	74.1	72.4	73.3	71.2

Chi-square		χ^2^(2,3079) = 13.03, p = 0.001	χ^2^(2,2536) = 23.10, p = 0.000	χ^2^(2,2154) = 36.10, p = 0.000	χ^2^(2,1797) = 28.12, p = 0.000	χ^2^(2,833) = 34.08, p = 0.000	χ^2^(2,3155) = 6.99, p = 0.030	χ^2^(2,2770) = 8.12, p = 0.017	χ^2^(2,2371) = 4.56, p = 0.102	χ^2^(2,2172) = 12.82, p = 0.002	χ^2^(2,721) = 8.27, p = 0.016

Over	1992	13.5	13.1	11.3	15.1	14.6	6.4	9.0	9.0	9.0	12.4
	2004	17.9	18.9	17.8	22.0	22.3	12.1	13.2	13.4	19.1	16.8
	2007	20.2	19.7	19.5	24.0	27.6	12.0	13.3	15.9	15.2	19.5

Chi-square		χ^2^(2,3079) = 9.17, p = 0.010	χ^2^(2,2536) = 10.13, p = 0.006	χ^2^(2,2154) = 15.37, p = 0.000	χ^2^(2,1797) = 13.16, p = 0.001	χ^2^(2,833) = 13.28, p = 0.001	χ^2^(2,3155) = 11.32, p = 0.003	χ^2^(2,2770) = 7.38, p = 0.025	χ^2^(2,2371) = 14.07, p = 0.001	χ^2^(2,2127) = 24.13, p = 0.000	χ^2^(2,721) = 4.57, p = 0.102

Obese	1992	2.9	1.7	1.5	2.1	0.8	4.4	3.0	3.0	2.7	2.6
	2004	5.8	5.9	5.0	6.6	6.5	3.1	3.6	5.1	4.7	9.2
	2007	5.3	6.2	6.4	5.0	8.5	2.7	4.2	4.2	4.7	6.3

Chi-square		χ^2^(2,3079) = 4.95, p = 0.084	χ^2^(2,2536) = 14.85, p = 0.001	χ^2^(2,2154) = 16.99, p = 0.000	χ^2^(2,1797) = 11.09, p = 0.004	χ^2^(2,833) = 16.07, p = 0.000	χ^2^(2,3155) = 3.22, p = 0.200	χ^2^(2,2770) = 1.60, p = 0.449	χ^2^(2,2371) = 3.47, p = 0.176	χ^2^(2,2127) = 3.88, p = 0.144	χ^2^(2,721) = 9.99, p = 0.007

**Table 5.** Mean BMI (kg/m^2^) within underweight, normal weight, overweight, and obesity among 14- and 15-year-old boys and girls in 1992, 1997, 2000, and 2006 (SD in parentheses).

		Boys		Girls	
BMI category	Year	14	15	14	15
Under	1992	16.0 (0.7)	16.5 (0.6)	16.3 (0.8)	16.8 (0.7)
	1997	15.7 (0.8)	16.4 (0.9)	16.3 (0.6)	16.7 (0.9)
	2000	15.9 (0.7)	16.3 (0.9)	16.4 (0.8)	16.7 (0.8)
	2006	15.8 (0.6)	16.2 (0.9)	16.2 (0.8)	16.6 (0.9)

Linear trend		*F* (1,3) = 2.32 p = 0.129	*F* (1,3) = 2.52 p = 0.114	*F* (1,3) = 0.29 p =.594	*F* (1,3) = 4.13 p = 0.043

Normal	1992	19.8 (1.5)	20.4 (1.5)	20.0(1.6)	20.5 (1.6)
	1997	19.7 (1.5)	20.4 (1.5)	19.9 (1.6)	20.5 (1.7)
	2000	20.0 (1.6)	20.6 (1.6)	19.9 (1.7)	20.5 (1.7)
	2006	19.9 (1.6)	20.6 (1.6)	20.1 (1.7)	20.5 (1.7)

Linear trend		*F* (1,3) = 6.49 p = 0.011	*F* (1,3) = 12.78 p = 0.000	*F* (1,3) = 2.63 p = 0.105	*F* (1,3) = 0.18 p = 0.669

Over	1992	24.7 (1.2)	25.3 (1.3)	25.4 (1.3)	25.9 (1.3)
	1997	24.6 (1.4)	25.0 (1.2)	25.4 (1.4)	26.0 (1.4)
	2000	24.7 (1.4)	25.4 (1.4)	25.4 (1.3)	25.9 (1.3)
	2006	24.8 (1.3)	25.4 (1.4)	25.3 (1.4)	26 0.0 (1.3)

Linear trend		*F* (1,3) = 1.14 p = 0.287	*F* (1,3) = 2.72 p = 0.100	*F* (1.3) = 0.21 p = 0.644	*F* (1,3) = 0.45 p = 0.505

Obese	1992	30.6 (1.4)	30.0 (1.2)	31.4 (1.6)	31.2 (1.4)
	1997	30.9 (2.1)	30.3 (1.5)	30.7 (1.3)	30.7 (1.0)
	2000	30.6 (1.9)	30.8 (1.7)	30.7 (1.5)	31.2 (1.5)
	2006	30.8 (2.1)	30.9 (1.8)	31.3 (1.5)	31.2 (1.5)

Linear trend		*F* (1,3) = 0.17 p = 0.684	*F* (1,3) = 5.20 p = 0.024	*F* (1.3) = 0.07 p = 0.793	*F* (1.3) = 0.17 p = 0.680

**Table 6.** Mean BMI (kg/m^2^) within underweight, normal weight, overweight, and obesity among 16- to 20-year-old boys in 1992, 2004, and 2007 (SD in parentheses).

Boys BMI category	Year	Age				
		16	17	18	19	20
Under	1992	17.0 (0.7)	17.2 (0.8)	17.5 (0.8)	17.7 (0.6)	N/A
	2004	16.3 (1.1)	17.1 (1.1)	17.6 (0.9)	17.2 (1.5)	17.6 (0.7)
	2007	16.3 (1.3)	16.6 (1.5)	17.5 (1.0)	17.3 (1.4)	16.6 (0.9)

Linear trend		*F* (1,2) = 3.59 p = 0.061	*F* (1,2) = 2.36 p = 0.128	*F* (1.2) = 0.00 p = 0.948	*F* (1,2) = 0.57 p = 0.456	*F* (1,2) = 1.92 p = 0.193

Normal	1992	21.1 (1.5)	21.4 (1.6)	22.0 (1.5)	22.0 (1.5)	22.5 (1.5)
	2004	20.9 (1.6)	21.5 (1.6)	22.0 (1.7)	22.2 (1.6)	22.1 (1.7)
	2007	21.0 (1.5)	21.6 (1.6)	22.0 (1.7)	22.3 (1.6)	22.3 (1.7)

Linear trend		*F* (1,2) = 0.84 p = 0.356	*F* (1,2) = 3.75 p = 0.053	*F* (1,2) = 0.04 p = 0.842	*F* (1,2) = 4.33 p = 0.038	*F* (1,2) = 4.38 p = 0.037

Over	1992	25.2 (1.3)	26.1 (1.4)	27.0 (1.4)	26.6 (1.3)	26.4 (1.2)
	2004	25.8 (1.3)	26.3 (1.4)	26.8 (1.4)	26.7 (1.3)	26.9 (1.2)
	2007	25.6 (1.2)	26.3 (1.3)	26.9 (1.4)	26.9 (1.4)	27.0 (1.3)

Linear trend		*F* (1,2) = 4.79 p = 0.029	*F* (1,2) = 1.16 p = 0.281	*F* (1,2) = 0.25 p = 0.619	*F* (1,2) = 2.12 p = 0.146	*F* (1,2) = 5.45 p = 0.021

Obese	1992	31.0 (2.1)	31.3 (1.3)	32.1 (2.4)	32.1 (2.4)	31.0 (0.7)
	2004	32.3 (2.9)	32.1 (2.4)	33.2 (2.7)	33.3 (2.1)	32.8 (2.0)
	2007	32.1 (3.1)	33.3 (2.8)	33.2 (2.5)	32.6 (2.7)	32.7 (2.2)

Linear trend		*F* (1,2) = 1.29 p = 0.258	*F* (1,2) = 6.68 p = 0.011	*F* (1,2) = 1.03 p = 0.312	*F* (1,2) = 0.27 p = 0.608	*F* (1,2) = 1.00 p = 0.323

N/A = there were no 20-year-old boys underweight in 1992.

**Table 7.** Mean BMI (kg/m^2^) within underweight, normal weight, overweight, and obesity among 16- to 20-year-old girls in 1992, 2004, and 2007.

Girls BMI category	Year	Age				
		16	17	18	19	20
Under	1992	17.1 (0.7)	17.5 (0.6)	17.7 (0.7)	17.7 (0.6)	17.7 (0.8)
	2004	17.0 (0.8)	17.4 (0.7)	17.4 (1.1)	17.6 (0.8)	18.1 (0.3)
	2007	16.9 (1.0)	17.3 (0.8)	17.6 (0.8)	17.5 (1.1)	17.0 (1.2)

Linear trend		*F* (1,2) = 1,59 p = 0.208	*F* (1,2) = 1.55 p = 0.215	*F* (1,2) = 1.32 p = 0.252	*F* (1,2) = 1.62 p = 0.205	*F* (1,2) = 0.01 p = 0.917

Normal	1992	20.7 (1.7)	21.0 (1.6)	21.4 (1.6)	21.5 (1.6)	21.5 (1.7)
	2004	20.8 (1.7)	21.2 (1.7)	21.5 (1.7)	21.6 (1.7)	21.7 (1.8)
	2007	20.9 (1.7)	21.4 (1.7)	21.6 (1.7)	21.7 (1.6)	21.6 (1.7)

Linear trend		*F* (1,2) = 2.27 p = 0.132	*F* (1,2) = 10.52 p = 0.001	*F* (1,2) = 5.58 p = 0.018	*F* (1,2) = 5.90 p = 0.015	*F* (1,2) = 0.39 p = 0.550

Over	1992	26.1 (1.4)	26.0 (1.2)	26.6 (1.3)	27.0 (1.2)	26.7 (1.4)
	2004	26.3 (1.3)	26.6 (1.4)	26.7 (1.2)	26.9 (1.4)	27.0 (1.3)
	2007	26.4 (1.4)	26.5 (1.4)	26.8 (1.3)	26.9 (1.4)	26.7 (1.4)

Linear trend		*F* (1,2) = 1.82 p = 0.178	*F* (1,2) = 6.11 p = 0.014	*F* (1,2) = 0.85 p = 0.356	*F* (1,2) = 0.13 p = 0.718	*F* (1,2) = 0.12 p = 0.730

Obese	1992	33.9 (2.9)	33.7 (2.7)	34.2 (2.5)	34.9 (2.3)	31.3 (1.2)
	2004	32.6 (2.8)	33.6 (3.1)	32.7 (2.3)	33.5 (2.6)	33.9 (2.9)
	2007	32.8 (2.9)	32.8 (2.7)	33.3 (2.7)	33.1 (2.7)	34.3 (3.4)

Linear trend		*F* (1,2) = 2.16 p = 0.145	*F* (1,2) = 0.99 p = 0.322	*F* (1,2) = 1.83 p = 0.180	*F* (1,2) = 4.28 p = 0.042	*F* (1,2) = 5.44 p = 0.025
